# SUMOylation‐Dependent Repression of BIRC7 by DNMT3a Suppresses Papillary Thyroid Carcinoma Progression

**DOI:** 10.1002/cbf.70290

**Published:** 2026-08-25

**Authors:** Kunpeng Liu, Zijie Su, Hongguang Li

**Affiliations:** ^1^ Department of Thyroid Surgery, Henan Provincial People's Hospital Zhengzhou University People's Hospital Zhengzhou China

**Keywords:** BIRC7, DNMT3a, papillary thyroid carcinoma

## Abstract

Papillary thyroid carcinoma (PTC) is the most prevalent endocrine malignancy, and while most patients respond well to standard therapies, a subset displays aggressive behavior, including lymph node metastasis and recurrence. The molecular mechanisms underlying PTC progression remain incompletely understood. Here, we identify baculoviral IAP repeat‐containing 7 (BIRC7) as a pro‐oncogenic factor upregulated in PTC tissues, particularly in metastatic tumors. Bioinformatic analysis of The Cancer Genome Atlas (TCGA) dataset and validation in clinical samples revealed a strong inverse correlation between BIRC7 expression and DNA methylation levels at its promoter. Mechanistically, we demonstrate that the DNA methyltransferase DNMT3a directly binds to the BIRC7 promoter and represses its transcription via promoter methylation. Pharmacological inhibition or siRNA‐mediated knockdown of DNMT3a leads to hypomethylation and transcriptional activation of BIRC7. Importantly, we show that DNMT3a undergoes SUMOylation, which negatively regulates its binding to the BIRC7 promoter. Inhibition of SUMOylation enhances DNMT3a chromatin occupancy and reduces BIRC7 expression. Functionally, BIRC7 promotes PTC cell proliferation and migration, while DNMT3a overexpression reverses these phenotypes in vitro and suppresses BIRC7‐mediated tumor growth in vivo. Our study uncovers a previously unrecognized SUMOylation‐dependent epigenetic regulatory axis wherein DNMT3a represses BIRC7 transcription. These findings establish BIRC7 as an epigenetically regulated oncogene in PTC and suggest that modulating DNMT3a SUMOylation may represent a novel therapeutic strategy for aggressive PTC.

## Summary

This study identifies BIRC7 as a novel epigenetically regulated oncogene driving papillary thyroid carcinoma progression, particularly lymph node metastasis. We uncover a previously unrecognized SUMOylation‐dependent DNMT3a–BIRC7 regulatory axis, in which DNMT3a directly represses BIRC7 transcription through promoter methylation. Crucially, DNMT3a SUMOylation is required for its chromatin binding and repressive function, linking post‐translational modification to DNA methylation control. Disruption of DNMT3a or its SUMOylation activates BIRC7 and enhances tumor aggressiveness, whereas inhibition of DNMT3a SUMOylation represses BIRC7 expression. These findings reveal a new layer of epigenetic regulation in PTC and suggest targeting DNMT3a SUMOylation as a therapeutic strategy for aggressive disease.

## Significance

Our research elucidates a previously unrecognized epigenetic mechanism in papillary thyroid carcinoma. We show that BIRC7 is upregulated in metastatic PTC due to promoter hypomethylation. This process is governed by DNMT3a, whose transcriptional repressive function is tightly controlled by its SUMOylation status. By linking post‐translational modification (SUMOylation) with epigenetic silencing, this study provides new insights into tumor progression and identifies potential biomarkers and therapeutic vulnerabilities in aggressive PTC.

**Abbreviations**
BIRC7: baculoviral IAP repeat‐containing 7
PTC: papillary thyroid carcinoma

## 1 Introduction

Thyroid carcinoma is the most common endocrine malignancy, with its incidence steadily increasing over the past several decades [1, 2]. Among its histological subtypes, papillary thyroid carcinoma (PTC) accounts for approximately 85%–90% of cases and is generally associated with favorable clinical outcomes following surgical resection and radioiodine therapy [3]. However, a distinct subset of patients displays aggressive pathological features, including extrathyroidal extension, regional lymph node metastasis, and local recurrence. These cases present considerable clinical challenges and are often resistant to conventional therapies [4]. Understanding the molecular mechanisms that drive PTC progression and metastasis is thus critical for improving risk stratification and identifying potential therapeutic targets.

Baculoviral IAP repeat‐containing 7 (BIRC7), also known as livin, is a member of the inhibitor of apoptosis (IAP) family, which exerts anti‐apoptotic effects primarily through the inhibition of caspases [5, 6]. Aberrant overexpression of BIRC7 has been documented in a variety of malignancies, including melanoma, lung cancer, breast cancer, and colon cancer, and is often associated with tumor cell survival, chemoresistance, and poor clinical outcomes [7, 8]. Mechanistically, BIRC7 promotes cellular proliferation and inhibits apoptosis, in part by antagonizing caspase‐3, −7, and −9 activity [9, 10]. Although BIRC7 has been proposed as an oncogene in several tumor types, its expression pattern, regulatory mechanisms, and functional role in PTC remain largely undefined. Notably, it is unclear whether BIRC7 contributes to the aggressive phenotypes observed in a subset of PTC patients, or whether it is subject to transcriptional regulation by tumor‐associated epigenetic mechanisms.

Epigenetic regulation, particularly DNA methylation at gene promoter regions, plays a central role in modulating gene expression during development and carcinogenesis [11, 12, 13]. DNA methylation is established and maintained by the DNA methyltransferase (DNMT) family, which includes DNMT1, DNMT3a, and DNMT3b. Among these, DNMT3a is the primary de novo methyltransferase responsible for establishing new methylation patterns and has been implicated in silencing tumor suppressor genes or regulating oncogene activity in multiple cancers [14, 15, 16, 17]. In thyroid cancer, aberrant promoter hypomethylation of oncogenes and hypermethylation of tumor suppressor genes are increasingly recognized as key epigenetic events [18, 19, 20]. However, the precise upstream epigenetic mechanisms driving the aberrant activation of BIRC7 in PTC remain to be elucidated. Notably, our preliminary bioinformatic screening of The Cancer Genome Atlas (TCGA‐THCA) dataset revealed that among various dysregulated genes, the BIRC7 promoter region displayed one of the most prominent demethylation cascades, which strongly and inversely correlated with its marked upregulation in metastatic PTC. This striking feature designated BIRC7 as a compelling candidate for deeply investigating the loss of epigenetic control during PTC progression.

SUMOylation is a key post‐translational modification within the ubiquitin‐proteasome system, playing vital roles in protein homeostasis and signal transduction. Dysregulation of this process has been closely associated with tumorigenesis [21]. SUMOylation plays a pivotal role in diverse biological processes, including gene transcription, cell cycle regulation, DNA damage repair, and innate immune responses. Its dysregulation has been implicated in tumorigenesis by promoting metastasis, therapeutic resistance, and immune evasion [22]. Mechanistically, SUMOylation involves the covalent attachment of small ubiquitin‐like modifier (SUMO) proteins to specific lysine residues on target proteins through a tightly regulated enzymatic cascade [23]. Moreover, post‐translational modifications (PTMs) such as SUMOylation have emerged as critical regulators of DNMT enzymatic activity and chromatin targeting. SUMOylation, the covalent attachment of SUMO proteins to lysine residues of target proteins, is known to affect protein stability, localization, and DNA‐binding capacity. Several studies have shown that SUMOylation of DNMT3a enhances its ability to associate with specific chromatin loci and modulate gene expression [24]. Whether SUMOylation is involved in regulating DNMT3a function in PTC, particularly in relation to oncogene suppression, remains to be elucidated.

In this study, we provide compelling evidence that BIRC7 is significantly overexpressed in PTC tissues, particularly in tumors with lymph node metastasis, and that its expression is inversely correlated with promoter DNA methylation. Through integrative analyzes of public datasets and validation in clinical samples and cell lines, we demonstrate that BIRC7 promoter hypomethylation is a recurrent feature of PTC and is mechanistically linked to reduced DNMT3a activity. Importantly, we identify DNMT3a as a direct transcriptional repressor of BIRC7 and show that its repressive function is inhibited by SUMOylation. Pharmacological inhibition of SUMOylation promotes DNMT3a binding to the BIRC7 promoter and results in transcriptional repression of BIRC7. Functional assays confirm that DNMT3a overexpression counteracts BIRC7‐driven cell proliferation and migration, and in vivo studies further demonstrate that DNMT3a suppresses BIRC7‐mediated tumor growth in xenograft models. Together, our findings uncover a previously unrecognized SUMOylation‐dependent DNMT3a–BIRC7 epigenetic axis, and suggest that this regulatory pathway plays a pivotal role in modulating PTC aggressiveness. Targeting this axis may represent a novel therapeutic approach for the treatment of advanced or recurrent PTC.

## 2 Materials and Methods

### 2.1 Cell Culture and Reagents

Human thyroid cell lines used in this study included papillary thyroid carcinoma (PTC) cell lines BCPAP (female, thyroid carcinoma; RRID: CVCL_0159), TPC‐1 (male, thyroid carcinoma; RRID: CVCL_6298), K1 (female, thyroid carcinoma; RRID: CVCL_2537), and IHH4 (female, thyroid carcinoma; RRID: CVCL_0B74), the anaplastic thyroid carcinoma (ATC) cell line 8505C (female, thyroid carcinoma; RRID: CVCL_1054), and the normal human thyroid follicular epithelial cell line Nthy‐ori 3‐1 (female, thyroid follicular epithelium; RRID: CVCL_2659). All cell lines were obtained from the Cell Bank of the Chinese Academy of Sciences (Shanghai, China) and were originally established from human thyroid tissue. The cell lines were acquired by our laboratory in 2022. Cells were cultured in RPMI‐1640 or DMEM medium (Gibco, Cat#11875‐093, USA) supplemented with 10% fetal bovine serum (FBS; Gibco, Cat#10099141C, USA) and 1% penicillin‐streptomycin (Gibco, Cat#15140122, USA) at 37°C in a humidified incubator containing 5% CO_2_. All cell lines were authenticated by short tandem repeat (STR) profiling by the supplier and were confirmed to be free of misidentification or cross‐contamination according to available databases. Mycoplasma contamination was routinely tested using PCR‐based assays, and only mycoplasma‐negative cells were used for the experiments described in this study. Zebularine, a DNA methyltransferase inhibitor, was purchased from Selleck Chemicals (Cat# S7306, USA). The SUMOylation inhibitors ginkgolic acid (GA; Cat# G4505) and 2‐D08 (Cat# SML1053) were obtained from Sigma‐Aldrich (USA). 5‐Azacytidine (5‐AzaC) was purchased from Abcam (Cat# ab142744, UK).

### 2.2 Quantitative Real‐Time PCR (qRT‐PCR)

Total RNA was extracted using TRIzol Reagent (Invitrogen, Cat# 15596018, USA) and reverse‐transcribed using the PrimeScript RT reagent kit (Takara, Cat# RR037A, Japan). qPCR was performed with TB Green Premix Ex Taq II (Takara, Cat# RR820A, Japan) on a QuantStudio 5 Real‐Time PCR System (Thermo Fisher Scientific, USA). Gene expression was normalized to GAPDH using the 2^–ΔΔCt^ method. Primer sequences are provided in Table 1.

**Table 1:** Oligonucleotide sequences and target coordinates used in this study.

Gene	Application	Primer direction	Sequence (5′–3′)
BIRC7	RT‐qPCR	PCR primer F	GCTCTGAGGAGTTGCGTCTG
		PCR primer R	CACACTGTGGACAAAGTCTCTT
DNMT1	RT‐qPCR	PCR primer F	AGGCGGCTCAAAGATTTGGAA
		PCR primer R	GCAGAAATTCGTGCAAGAGATTC
DNMT3A	RT‐qPCR	PCR primer F	CCGATGCTGGGGACAAGAAT
		PCR primer R	CCCGTCATCCACCAAGACAC
DNMT3B	RT‐qPCR	PCR primer F	AGGGAAGACTCGATCCTCGTC
		PCR primer R	GTGTGTAGCTTAGCAGACTGG
GAPDH	RT‐qPCR	PCR primer F	GCACCGTCAAGGCTGAGAAC
		PCR primer R	GCCTTCTCCATGGTGGTGAA
BIRC7 promoter	MeDIP	PCR primer F	GCTCCGACCTCTCCTACTTC
		PCR primer R	CGCAGCTTCCTTTTCCAAGG
BIRC7 MSP (M)	Methylation‐specific PCR	PCR primer F	GTTTTCGTTGTCGTTATCGTC
		PCR primer R	CGAAATCTAAACGACGAACG
BIRC7 MSP (U)	Methylation‐specific PCR	PCR primer F	GTTTTTTGTTGTTGTTATTGTTG
		PCR primer R	CAAAATCTAAACAACAAACAAAC
pGL3‐BIRC7		PCR primer F	CGGGGTACCGCAGAGACCCCAGTGACAG
		PCR primer R	CCGCTCGAGCGGTCACCCTGGTATCTTGAC

### 2.3 Western Blot Analysis

Cells were lysed in RIPA buffer (Beyotime, Cat# P0013B, China) containing protease and phosphatase inhibitors (Thermo Fisher Scientific, Cat# 78442, USA). Protein concentration was determined using the BCA Protein Assay Kit (Thermo Fisher Scientific, Cat# 23227, USA). Equal amounts of protein were separated by SDS‐PAGE and transferred to PVDF membranes (Millipore, Cat# IPVH00010, USA). Membranes were blocked with 5% non‐fat milk and incubated with primary antibodies overnight at 4°C. The following antibodies were used: BIRC7 (Abcam, Cat# ab97304, UK), DNMT3a (Cell Signaling Technology, Cat# 3598, USA), DNMT1 (Cell Signaling Technology, Cat# 5032, USA), DNMT3b (Cell Signaling Technology, Cat# 57836, USA), SUMO1 (Abcam, Cat# ab32058, UK), and GAPDH (Proteintech, Cat# 60004‐1‐Ig, China). HRP‐conjugated secondary antibodies (Abcam, Cat# ab6721 and ab6728, UK) were used, and signals were visualized using an enhanced chemiluminescence (ECL) detection system (Thermo Fisher Scientific, Cat# 32106, USA).

### 2.4 Methylated DNA Immunoprecipitation (MeDIP)‐qPCR

Genomic DNA was extracted using the DNeasy Blood & Tissue Kit (Qiagen, Cat# 69504, Germany). DNA was sheared to 200–600 bp by sonication and subjected to immunoprecipitation using anti‐5‐methylcytosine antibody (Abcam, Cat# ab10805, UK) and Dynabeads Protein G (Invitrogen, Cat# 10003D, USA). DNA was purified and analyzed by qPCR using primers targeting the BIRC7 promoter region.

### 2.5 Methylation‐Specific PCR (MSP)

Genomic DNA was bisulfite‐converted using the EZ DNA Methylation‐Gold Kit (Zymo Research, Cat# D5005, USA). MSP was performed using methylation‐specific and unmethylation‐specific primers for the BIRC7 promoter region. PCR products were resolved on 2% agarose gels and visualized with GelRed (Biotium, Cat# 41003, USA).

### 2.6 siRNA Transfection

siRNAs targeting DNMT1, DNMT3a, and DNMT3b were purchased from GenePharma (Shanghai, China). Cells were transfected using Lipofectamine RNAiMAX (Invitrogen, Cat# 13778030, USA) according to the manufacturer's protocol. Knockdown efficiency was validated by qRT‐PCR and Western blot 48 h post‐transfection.

### 2.7 Chromatin Immunoprecipitation (ChIP)‐qPCR

ChIP was performed using the SimpleChIP Enzymatic Chromatin IP Kit (Cell Signaling Technology, Cat# 9003, USA). Briefly, cells were crosslinked with 1% formaldehyde, lysed, and chromatin was sheared enzymatically. Immunoprecipitation was performed using anti‐DNMT3a antibody or IgG control. DNA was purified and analyzed by qPCR with primers targeting the BIRC7 promoter region.

### 2.8 Dual‐Luciferase Reporter Assay

The BIRC7 promoter region was cloned into the pGL3‐Basic vector (Promega, Cat# E1751, USA). Luciferase reporter plasmids were co‐transfected with Renilla luciferase control (pRL‐TK) into cells using Lipofectamine 3000 (Invitrogen, Cat# L3000008, USA). Luciferase activity was measured 48 h later using the Dual‐Luciferase Reporter Assay System (Promega, Cat# E1910, USA) and normalized to Renilla activity.

### 2.9 Cell Proliferation and Migration Assays

Cell proliferation was assessed using the Cell Counting Kit‐8 (CCK‐8; Dojindo, Cat# CK04, Japan) according to the manufacturer's instructions. Absorbance at 450 nm was measured using a microplate reader (BioTek, USA).

Migration assays were performed using 24‐well Transwell chambers with 8 µm pores (Corning, Cat# 3422, USA). Cells (1 × 10^5^) in serum‐free medium were seeded into the upper chambers, while medium with 10% FBS was added to the lower chambers. After 24 h, migrated cells were fixed with methanol, stained with crystal violet (Beyotime, Cat# C0121, China), and counted under a microscope.

### 2.10 In Vivo Xenograft Model

All animal experiments were approved by the Institutional Animal Care and Use Committee and conducted in compliance with institutional and national guidelines for the care and use of laboratory animals. Male BALB/c nude mice (6 weeks old) were purchased from the Shanghai SLAC Laboratory Animal Co. Ltd (China). After 1 week of acclimation, the mice were randomly divided into 4 groups (*n* = 5 per group). BCPAP cells stably overexpressing BIRC7, DNMT3a, or both were injected subcutaneously (2.5 × 10^6^ cells/mouse). Tumor volume was measured every 5 days using calipers and calculated as: volume = (length × width^2^)/2. At the end of the 30‐day observation period, mice were euthanized by CO_2_ inhalation followed by cervical dislocation, in accordance with the American Veterinary Medical Association (AVMA) Guidelines for the Euthanasia of Animals (2020). Tumors were then collected and weighed.

### 2.11 Statistical Analysis

The data were expressed as mean ± standard deviation. A Student's t‐test was applied to compare between two groups, while a one‐way analysis of variance followed by Tukey's post hoc test was utilized for comparisons involving more than two groups. Statistical significance was identified when *p* < 0.05. All statistical analyzes were conducted using GraphPad Prism (version 8.0).

## 3 Results

### 3.1 BIRC7 Is Upregulated in PTC and Is Associated With Reduced Promoter Methylation

**Figure 1:**
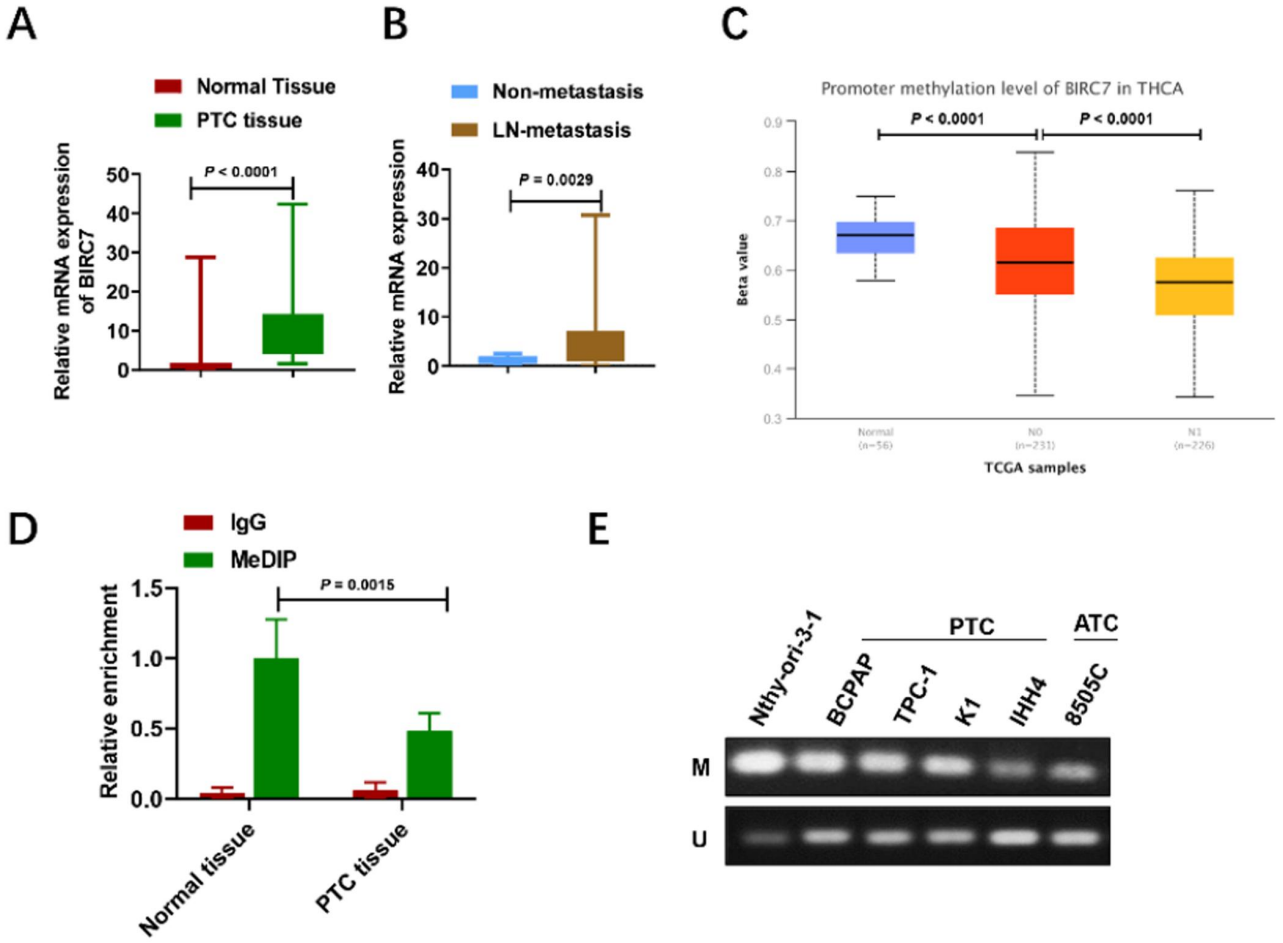
BIRC7 is upregulated in papillary thyroid carcinoma (PTC) and associated with decreased promoter DNA methylation. A, Quantitative real‐time PCR (qRT‐PCR) analysis reveals significantly elevated *BIRC7* mRNA levels in PTC tissues compared to paired adjacent normal tissues (*n* = 48 pairs, *p* < 0.0001). B, *BIRC7* mRNA expression is significantly higher in PTC tissues with lymph node (LN) metastasis (*n* = 31) than in those without metastasis (*n* = 17) (*p* = 0.0029). C, Analysis of *BIRC7* promoter methylation using the TCGA dataset. DNA methylation levels in normal thyroid tissues (Normal, *n* = 56), non‐metastatic PTC (N0, *n* = 231), and metastatic PTC (N1, *n* = 226) were compared; a progressive and significant decrease in promoter methylation was observed from Normal to N0, and from N0 to N1 (both *p* < 0.0001). D, Methylated DNA immunoprecipitation (MeDIP)‐qPCR analysis of the *BIRC7* promoter region in paired PTC and adjacent normal tissues. Significantly reduced DNA methylation was detected in tumor tissues compared to normal tissues (*p* = 0.0015). E, Methylation‐specific PCR (MSP) was used to assess *BIRC7* promoter methylation status in the normal thyroid cell line (Nthy‐ori‐3‐1), PTC cell lines (BCPAP, TPC‐1, K1, IHH4), and anaplastic thyroid cancer cell line (8505 C) after zebularine treatment for 3 days. Compared to Nthy‐ori‐3‐1, PTC and ATC cell lines exhibited reduced methylation of the *BIRC7* promoter (M, methylated; U, unmethylated). Data are presented as mean ± SD of *n* = 3 independent experiments.

To investigate the clinical relevance of BIRC7 in papillary thyroid carcinoma (PTC), we first assessed its expression levels in human PTC tissues. Quantitative RT‐PCR analysis revealed a significant upregulation of BIRC7 mRNA in tumor samples compared to matched adjacent non‐tumor tissues (Figure 1A). Moreover, stratification of PTC specimens based on lymph node metastasis status showed that BIRC7 expression was markedly elevated in metastatic tumors relative to non‐metastatic counterparts (Figure 1B), suggesting a potential role of BIRC7 in promoting PTC progression and dissemination.

To explore the regulatory mechanism underlying BIRC7 dysregulation, we performed bioinformatic analysis of TCGA datasets and observed a stepwise decline in DNA methylation levels at the BIRC7 promoter across normal thyroid tissues, non‐metastatic PTC (N0), and metastatic PTC (N1), with the lowest methylation levels observed in N1 tumors (Figure 1C).

To achieve higher, CpG‐resolved mapping of this epigenetic alteration, we systematically analyzed the Illumina HM450K array data from the TCGA‐THCA cohort (507 tumors vs. 56 normal tissues). Genomic mapping of 13 specific CpG probes across the *BIRC7* locus identified a distinct cluster of 8 CpGs located within the precise promoter region (Supporting Information Figure S1A). Strikingly, 7 out of these 8 promoters CpGs exhibited highly significant hypomethylation in PTC tumors at the single‐nucleotide level (FDR < 0.05, Supporting Information Figure S1B). Furthermore, correlation analysis demonstrated that the methylation levels (β‐values) of these specific promoters CpGs were strongly and negatively correlated with *BIRC7* mRNA expression (Supporting Information Figure S1C). This inverse correlation between high‐resolution promoter methylation and *BIRC7* expression strongly suggested an epigenetic regulatory mechanism. To validate these large‐cohort, CpG‐level observations experimentally, we performed methylated DNA immunoprecipitation followed by qPCR (MeDIP‐qPCR) using paired clinical samples and found a significant reduction in DNA methylation at the BIRC7 promoter region in PTC tissues compared to adjacent non‐tumor tissues (Figure 1D). Further, we examined BIRC7 promoter methylation in thyroid cancer cell lines. Following 3‐day treatment with zebularine, a DNA methyltransferase inhibitor, methylation‐specific PCR (MSP) analysis revealed that BIRC7 promoter methylation was markedly lower in PTC cell lines (BCPAP, TPC‐1, K1, and IHH4) and the ATC cell line 8505C than in the normal thyroid epithelial cell line Nthy‐ori‐3‐1 (Figure 1E). These findings collectively suggest that hypomethylation of the BIRC7 promoter contributes to its transcriptional upregulation in PTC.

### 3.2 DNMT3a Suppresses BIRC7 Expression Through Promoter Methylation

**Figure 2:**
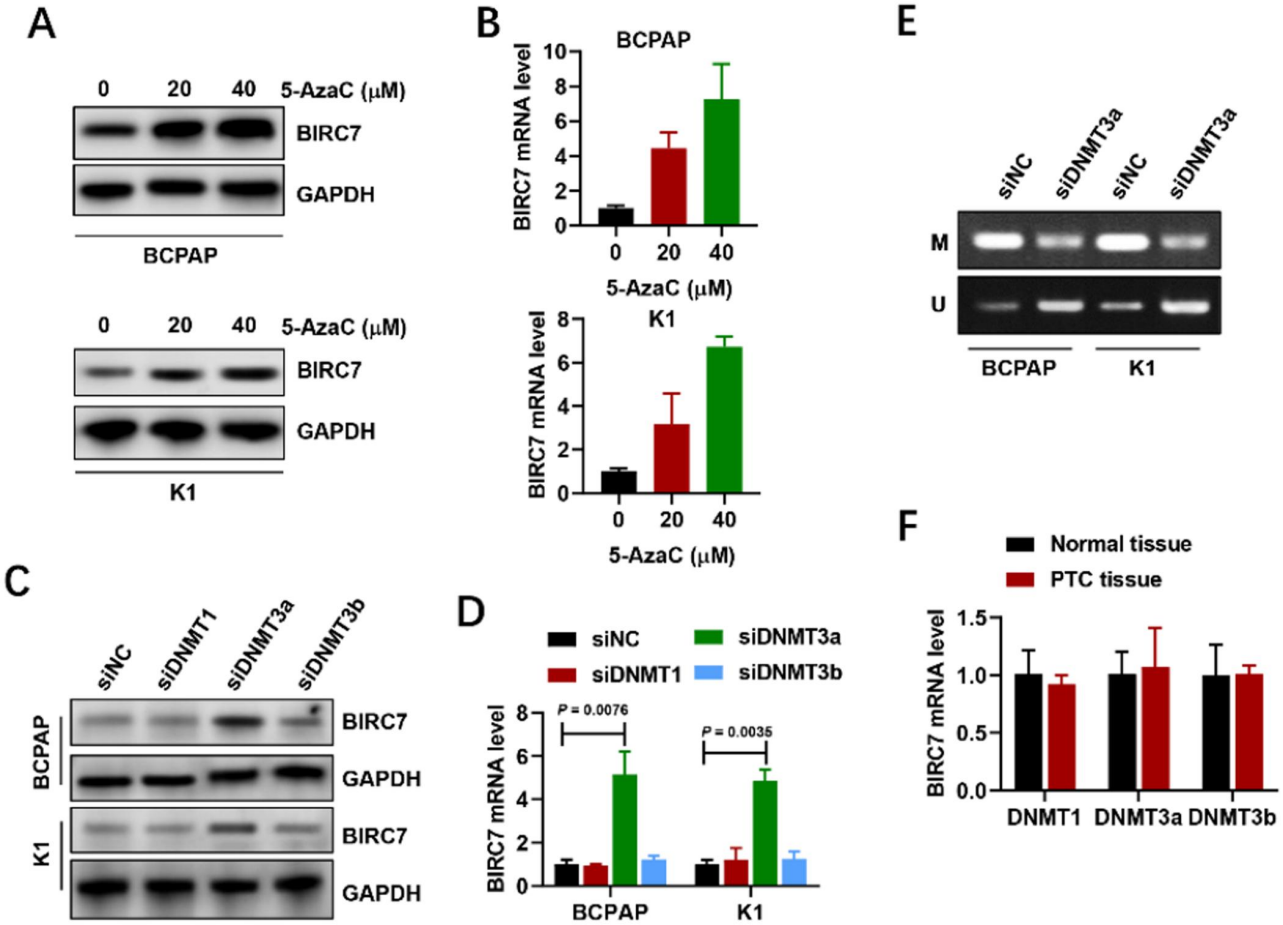
DNMT3a suppresses BIRC7 expression through promoter methylation. A, Western blot analysis of BIRC7 protein levels in BCPAP and K1 cells treated with increasing concentrations (0, 20, and 40 µM) of the DNA methylation inhibitor 5‐AzaC for 48 h. GAPDH was used as a loading control. Representative images of three independent experiments are shown. B, Quantitative real‐time PCR (qRT‐PCR) analysis of *BIRC7* mRNA levels following 24‐h 5‐AzaC treatment in BCPAP and K1 cells. Data are presented as mean ± SD of *n* = 3 independent experiments. C, Western blot analysis of BIRC7 protein levels after siRNA‐mediated knockdown of DNMT1, DNMT3a, or DNMT3b (siDNMT1, siDNMT3a, siDNMT3b) compared to the negative control (siNC) in BCPAP and K1 cells. Representative images of three independent experiments are shown. D, qRT‐PCR detection of *BIRC7* mRNA expression following knockdown of DNMTs as in (C). Knockdown of DNMT3a significantly increased *BIRC7* mRNA levels compared to the siNC group in both BCPAP (*p* = 0.0076) and K1 (*p* = 0.0035) cells. Data are presented as mean ± SD of *n* = 3 independent experiments. E, Methylation‐specific PCR (MSP) analysis showing demethylation of the *BIRC7* promoter after DNMT3a knockdown in BCPAP and K1 cells (M, methylated; U, unmethylated). Representative images of three independent experiments are shown. F, Expression levels of *DNMT1*, *DNMT3a*, and *DNMT3b* in paired PTC and normal thyroid tissues determined by qRT‐PCR; no significant differences were observed between the groups. Data are presented as mean ± SD (*n* = 15 paired tissues).

We next sought to identify the DNA methyltransferase responsible for BIRC7 promoter methylation. Treatment of BCPAP and K1 cells with increasing concentrations of 5‐AzaC led to a dose‐dependent upregulation of BIRC7 protein expression as measured by Western blot (Figure 2A), and a corresponding increase in mRNA levels as determined by qRT‐PCR (Figure 2B), implicating DNA methylation as a key suppressive mechanism.

To identify the specific DNMT involved, we performed siRNA‐mediated knockdown of DNMT1, DNMT3a, and DNMT3b in both BCPAP and K1 cells. Notably, only knockdown of DNMT3a robustly increased BIRC7 protein (Figure 2C) and mRNA (Figure 2D) levels, implicating DNMT3a as the major suppressor of BIRC7. MSP analysis further confirmed that silencing DNMT3a led to promoter demethylation of BIRC7 in both cell lines (Figure 2E). Interestingly, when the expression levels of DNMT1, DNMT3a, and DNMT3b were examined in PTC patient tissues and adjacent normal thyroid tissues, no significant differences were observed (Figure 2F), indicating that DNMT3a suppression of BIRC7 is likely due to post‐translational modulation rather than altered transcription.

### 3.3 SUMOylation Regulates DNMT3a Activity and Indirectly Modulates BIRC7 Expression

**Figure 3:**
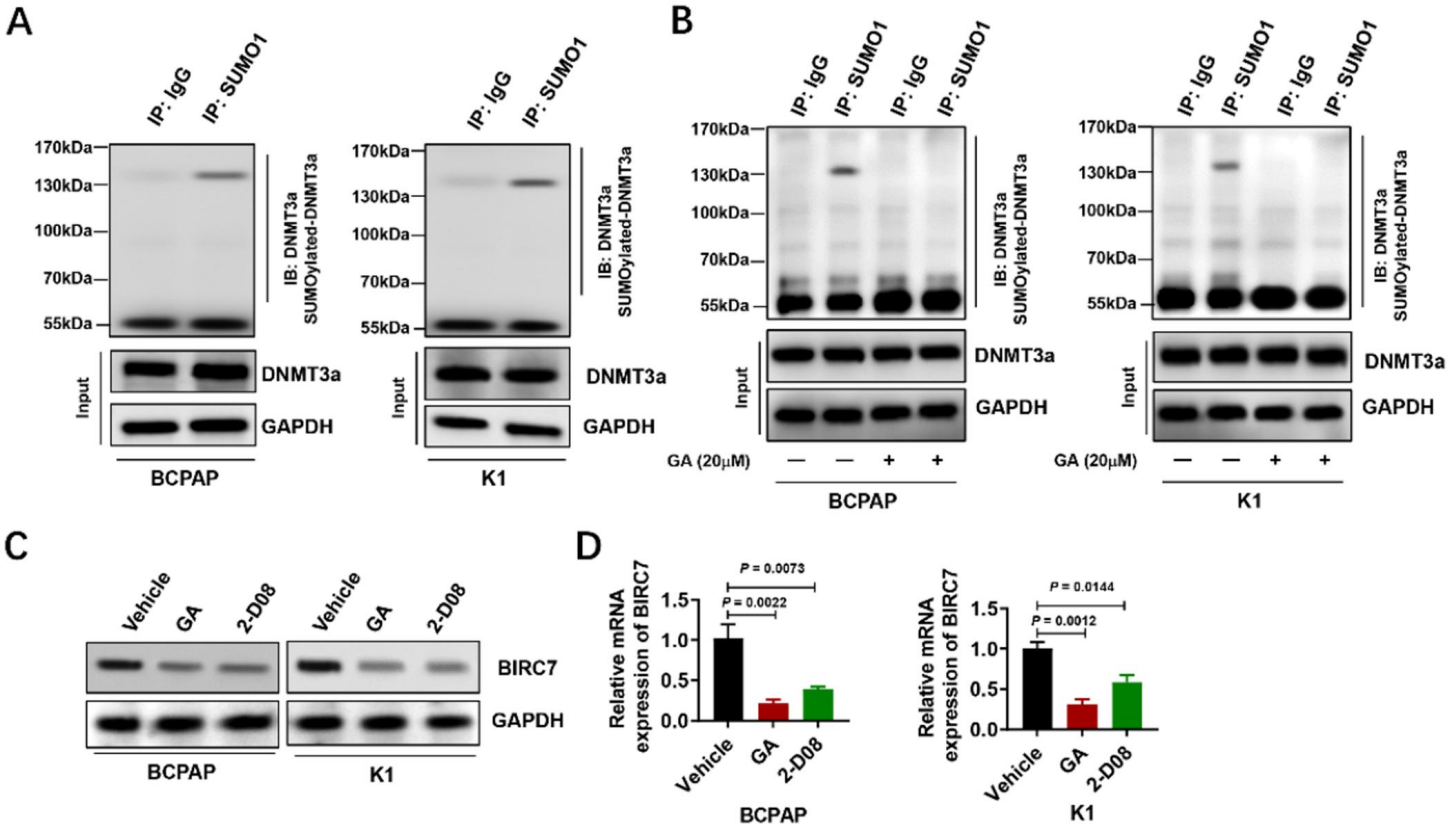
SUMOylation modulates DNMT3a enzymatic activity and affects BIRC7 expression. A, Co‐immunoprecipitation (Co‐IP) of endogenous DNMT3a in BCPAP and K1 cell lysates using a SUMO1 antibody, followed by immunoblotting (IB) to detect SUMOylated DNMT3a. IgG was used as a negative control. Representative images of three independent experiments are shown. B, Co‐IP assays in BCPAP and K1 cells treated with or without the SUMOylation inhibitor ginkgolic acid (GA, 20 µM) showing reduced DNMT3a SUMOylation. Representative images of three independent experiments are shown. C, Western blot analysis of BIRC7 protein levels after treatment with vehicle, GA, or 2‐D08 in BCPAP and K1 cells; both inhibitors prominently reduced BIRC7 protein expression. GAPDH served as a loading control. Representative images of three independent experiments are shown. D, Quantitative real‐time PCR (qRT‐PCR) analysis of *BIRC7* mRNA levels in BCPAP and K1 cells treated with vehicle, GA, or 2‐D08. Both treatments significantly suppressed *BIRC7* transcription compared to the vehicle control in BCPAP (*p* = 0.0022 for GA; *p* = 0.0073 for 2‐D08) and K1 cells (*p* = 0.0012 for GA; *p* = 0.0144 for 2‐D08). Data are presented as mean ± SD of *n* = 3 independent experiments.

Given that DNMT3a transcript levels were unchanged in PTC tissues despite reduced BIRC7 promoter methylation, we hypothesized that DNMT3a enzymatic activity might be regulated post‐translationally. Co‐immunoprecipitation (Co‐IP) using SUMO1 antibodies followed by immunoblotting in BCPAP and K1 cell lysates revealed the presence of SUMOylated DNMT3a (Figure 3A), suggesting that DNMT3a is subject to SUMO modification.

To further explore this mechanism, cells were treated with the SUMOylation inhibitor ginkgolic acid (GA). Co‐IP analysis demonstrated that GA markedly reduced the SUMOylation of DNMT3a (Figure 3B), confirming that SUMO modification of DNMT3a is reversible and sensitive to chemical inhibition. To rule out potential off‐target effects associated with pharmacological inhibitors and to provide direct causal evidence, we genetically perturbed the SUMO pathway by knocking down the sole SUMO‐conjugating enzyme, UBC9, using small interfering RNA (siUBC9). Consistent with the inhibitor data, Co‐IP analysis revealed that UBC9 knockdown substantially diminished the SUMOylation of DNMT3a in both BCPAP and K1 cells (Supporting Information Figure S2A).

We next examined the impact of SUMOylation inhibition on BIRC7 expression. Western blot analysis showed that treatment with either GA or the SUMOylation inhibitor 2‐D08 significantly decreased BIRC7 protein levels in both BCPAP and K1 cells (Figure 3C). Crucially, this downregulation was completely phenocopied by genetic ablation; transient transfection with siUBC9 markedly reduced BIRC7 protein levels compared to the negative control (siNC) (Supporting Information Figure S2B). Consistent with this, qRT‐PCR analysis revealed that both chemical inhibitors and genetic UBC9 knockdown reduced *BIRC7* mRNA expression (Figure 3D and Supporting Information Figure S2C), indicating that SUMOylation enhances *BIRC7* transcription, likely through inhibiting the repressive function of DNMT3a.

### 3.4 DNMT3a Directly Binds to the BIRC7 Promoter and Represses its Transcription

**Figure 4:**
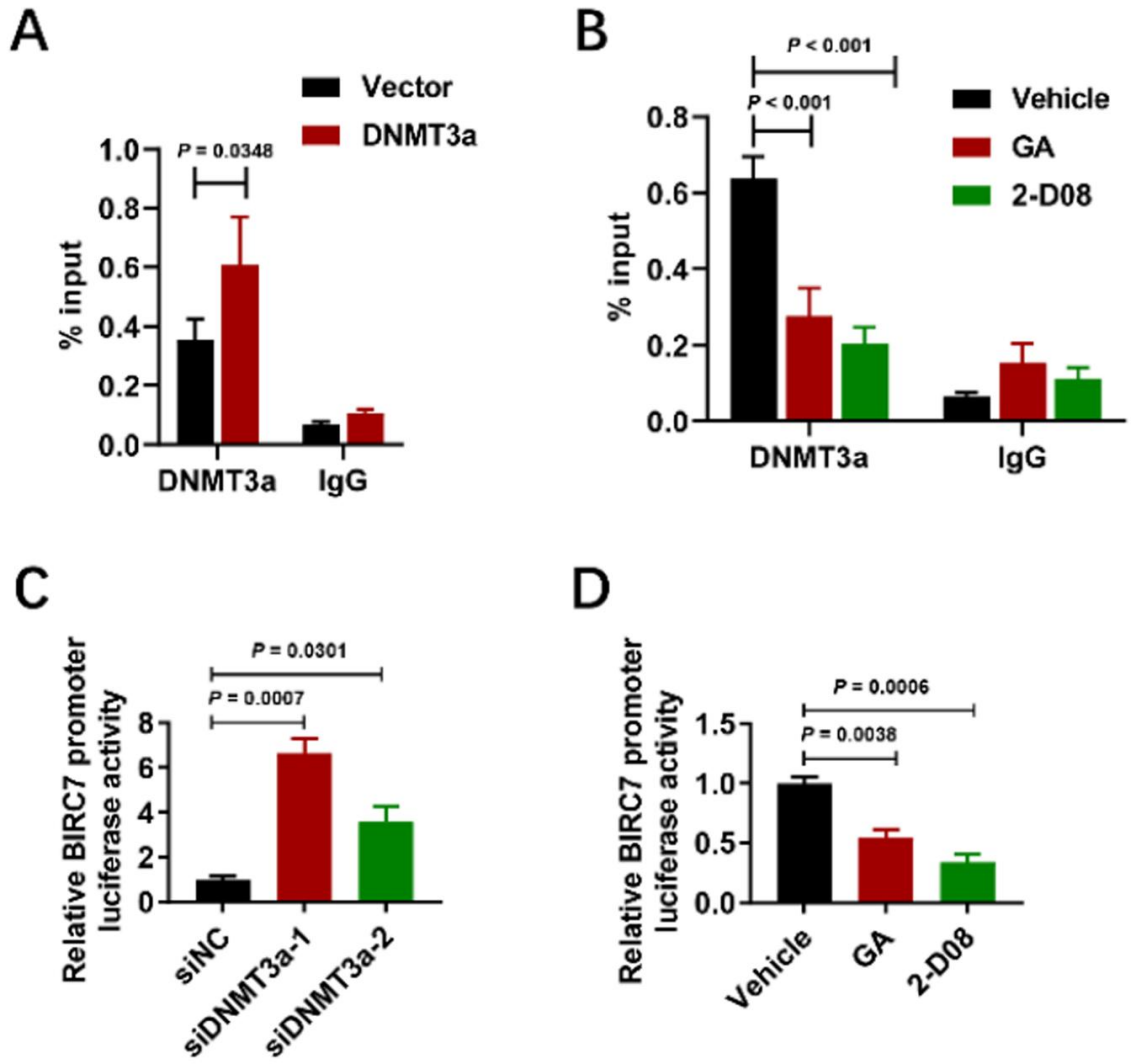
DNMT3a directly binds to the BIRC7 promoter and represses its transcription. A, Chromatin immunoprecipitation (ChIP)‐qPCR assay in BCPAP cells overexpressing DNMT3a or an empty vector, demonstrating significantly increased DNMT3a binding to the *BIRC7* promoter region in the overexpression group (*p* = 0.0348). IgG was used as a negative control. Data are presented as mean ± SD of *n* = 3 independent experiments. B, ChIP‐qPCR showing significantly decreased enrichment of DNMT3a at the *BIRC7* promoter after treatment with the SUMOylation inhibitors GA (*p* < 0.001) or 2‐D08 (*p* < 0.001) for 24 h in BCPAP cells compared to the vehicle control. IgG served as a negative pull‐down control. Data are presented as mean ± SD of *n* = 3 independent experiments. C, Dual‐luciferase reporter assay measuring *BIRC7* promoter activity in BCPAP cells with DNMT3a knockdown (siDNMT3a‐1 and siDNMT3a‐2) or a negative control (siNC). The results show significantly increased promoter activity upon DNMT3a depletion (*p* = 0.0007 for siDNMT3a‐1; *p* = 0.0301 for siDNMT3a‐2). Data are presented as mean ± SD of *n* = 3 independent biological replicates. D, Luciferase reporter assays demonstrating that GA or 2‐D08 treatment significantly suppresses *BIRC7* transcriptional activity in BCPAP cells compared to the vehicle control (*p* = 0.0038 for GA; *p* = 0.0006 for 2‐D08). Data are presented as mean ± SD of *n* = 3 independent experiments.

To confirm whether DNMT3a directly targets the BIRC7 promoter, chromatin immunoprecipitation followed by qPCR (ChIP‐qPCR) was performed in BCPAP cells overexpressing DNMT3a. The results demonstrated significant enrichment of DNMT3a at the BIRC7 promoter region (Figure 4A), supporting a direct interaction between DNMT3a and the BIRC7 gene locus.

Importantly, treatment with either GA or 2‐D08 significantly enhanced DNMT3a binding to the BIRC7 promoter, as shown by ChIP‐qPCR (Figure 4B), suggesting that SUMOylation is required for DNMT3a's chromatin association and gene‐suppressive function.

To evaluate the functional consequence of DNMT3a depletion on BIRC7 transcriptional activity, we performed dual‐luciferase reporter assays. Knockdown of DNMT3a significantly enhanced the transcriptional activity of the BIRC7 promoter (Figure 4C). Conversely, inhibition of SUMOylation by GA or 2‐D08 led to a notable suppression of BIRC7 promoter activity (Figure 4D), further supporting a model in which unSUMOylated DNMT3a represses BIRC7 expression by maintaining promoter methylation.

### 3.5 DNMT3a Counteracts BIRC7‐Mediated Proliferation and Migration in PTC Cells

**Figure 5:**
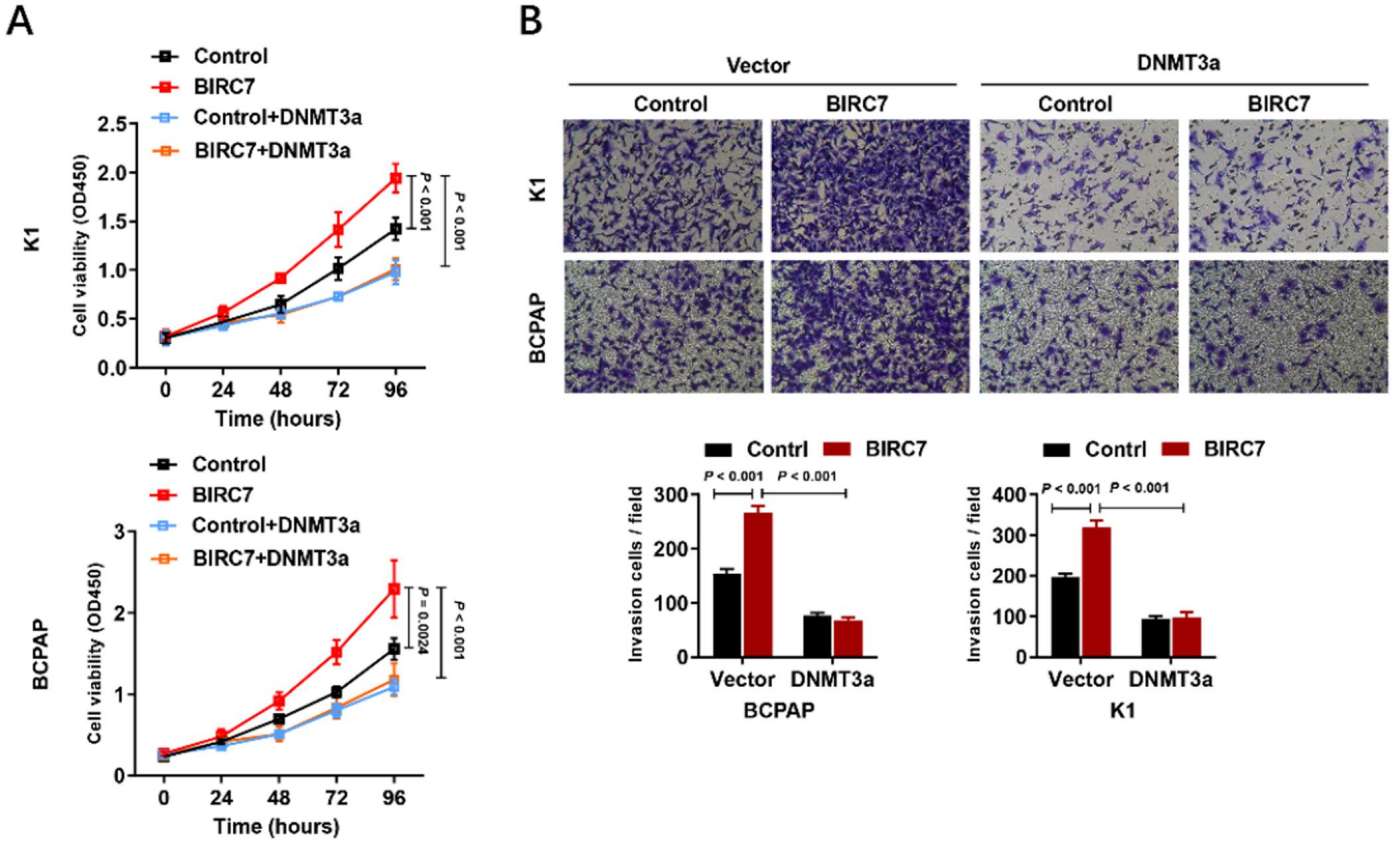
DNMT3a overexpression abrogates BIRC7‐induced proliferation and migration in PTC cells. A, Cell Counting Kit‐8 (CCK‐8) assays measuring the cell viability (proliferation) of K1 and BCPAP cells over 96 h following transfection with BIRC7 and/or DNMT3a. Overexpression of BIRC7 significantly promoted cell proliferation compared to the control group in both K1 (*p* < 0.001) and BCPAP (*p* = 0.0024) cells. This BIRC7‐induced proliferation was significantly abrogated by co‐transfection with DNMT3a (*p* < 0.001 for both K1 and BCPAP cells). Data are presented as mean ± SD of *n* = 3 independent experiments. B, Transwell invasion assays assessing cell invasive capacity following overexpression of BIRC7 and/or DNMT3a in K1 and BCPAP cells. Representative images of invaded cells are shown (top). Quantitative bar graphs (bottom) demonstrate that BIRC7 overexpression significantly increased the number of invading cells per field compared to the vector control (*p* < 0.001 for both BCPAP and K1 cells), whereas co‐overexpression of DNMT3a significantly reversed this effect (*p* < 0.001 for both BCPAP and K1 cells). Data are presented as mean ± SD of *n* = 3 independent experiments.

To determine the functional relevance of the DNMT3a‐BIRC7 regulatory axis in thyroid cancer biology, we conducted proliferation and migration assays in BCPAP and K1 cells co‐transfected with BIRC7 and/or DNMT3a. Overexpression of BIRC7 significantly enhanced cell proliferation as measured by CCK‐8 assays, while co‐expression of DNMT3a abrogated this effect (Figure 5A). Similarly, transwell migration assays revealed that BIRC7 promoted cell migration, whereas DNMT3a co‐expression markedly suppressed BIRC7‐driven motility (Figure 5B). To strictly establish the pathway order and reduce reliance on overexpression models, we performed complementary loss‐of‐function and epistasis experiments. As expected, knockdown of BIRC7 (shBIRC7) significantly suppressed cell proliferation and invasion. Conversely, silencing DNMT3a (siDNMT3a) enhanced these malignant phenotypes. Depletion of DNMT3a completely reversed the suppressive effects of BIRC7 knockdown, driving the cells back to a highly proliferative and invasive state (Supporting Information Figure S3A–B). These data support a tumor‐suppressive role for DNMT3a and confirm its ability to functionally antagonize BIRC7 in PTC cells.

### 3.6 DNMT3a Overexpression Suppresses BIRC7‐Driven Tumor Growth In Vivo

**Figure 6:**
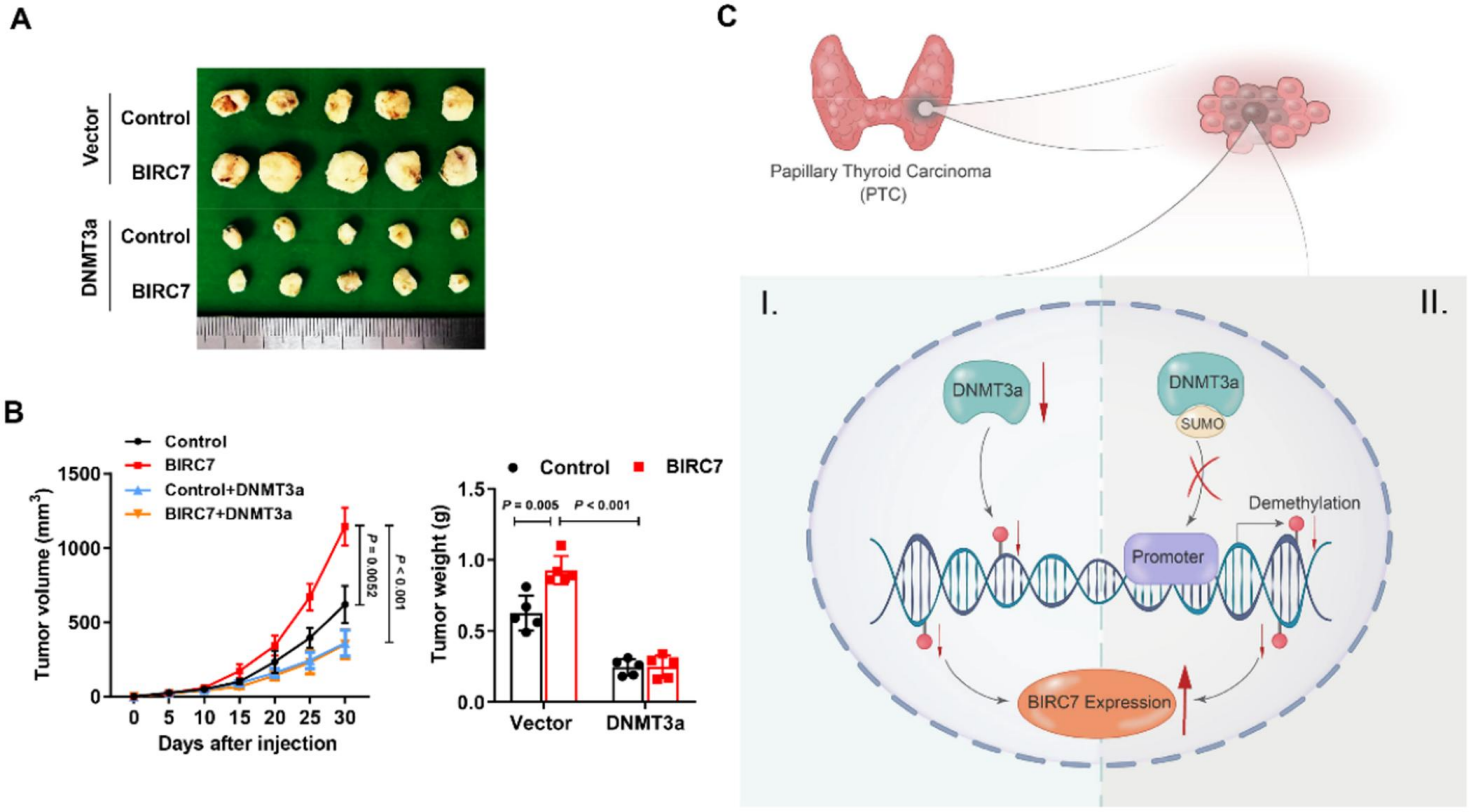
DNMT3a overexpression impairs BIRC7‐driven xenograft tumor growth in vivo. BCPAP cells stably overexpressing BIRC7 alone, DNMT3a alone, or both BIRC7 and DNMT3a (2.5 × 10^6^ cells per mouse) were subcutaneously injected into the right flank of male BALB/c nude mice (*n* = 5 mice per group). A, Representative images of the excised xenograft tumors harvested from each group at 30 days post‐injection. B, Tumor volume (left) was measured every 5 days to plot the growth curves, and tumor weights (right) were measured at the endpoint (day 30). Overexpression of BIRC7 significantly increased tumor volume (*p* = 0.0052) and tumor weight (*p* = 0.005) compared to the vector control group. Co‐expression of DNMT3a significantly inhibited the BIRC7‐induced increase in tumor volume (*p* < 0.001) and tumor weight (*p* < 0.001). Data are presented as mean ± SD of *n* = 5 biological replicates. C, Schematic diagram illustrating the proposed mechanism by which DNMT3a and its SUMOylation regulate BIRC7 expression in Papillary Thyroid Carcinoma (PTC). SUMOylation of DNMT3a impairs its ability to methylate the *BIRC7* promoter, leading to DNA demethylation and subsequent transcriptional upregulation of BIRC7, which ultimately promotes tumor progression.

To assess the in vivo significance of the DNMT3a–BIRC7 axis, we established xenograft models by subcutaneously injecting BCPAP cells stably overexpressing BIRC7 alone, DNMT3a alone, or both into male BALB/c nude mice. Mice injected with BIRC7‐expressing cells developed substantially larger tumors compared to controls, whereas co‐expression of DNMT3a significantly reduced tumor burden (Figure 6A–C). Tumor volume measurements over time demonstrated that DNMT3a overexpression effectively suppressed BIRC7‐induced tumor growth throughout the course of the experiment (Figure 6B), and final tumor weights were significantly lower in the DNMT3a+BIRC7 group compared to the BIRC7‐only group (Figure 6C). These results underscore the therapeutic potential of targeting DNMT3a SUMOylation in PTC.

## 4 Discussion

Epigenetic dysregulation has emerged as a defining hallmark of cancer, intricately modulating gene expression programs that underpin tumor initiation, progression, and therapeutic resistance [25, 26, 27]. In this study, we identify BIRC7, a member of the inhibitor of apoptosis (IAP) family, as a transcriptionally upregulated oncogene in PTC, driven by promoter hypomethylation. Importantly, we uncover a previously uncharacterized SUMOylation‐dependent regulatory axis, wherein DNMT3a suppresses BIRC7 expression via promoter methylation. This regulatory mechanism highlights a novel layer of epigenetic control over PTC aggressiveness and offers new insights into the interplay between post‐translational modifications and DNA methylation in thyroid carcinogenesis.

While BIRC7 has been implicated in anti‐apoptotic signaling in several malignancies‐including melanoma, colorectal cancer, and lung carcinoma‐its role in PTC has remained largely unexplored. Our findings extend its functional relevance to thyroid cancer and position BIRC7 not merely as a marker of malignancy but as a mechanistic driver of proliferation and metastatic potential. The observed association between high BIRC7 expression and lymph node metastasis underscores its potential utility as a prognostic indicator, particularly in identifying PTC patients with a propensity for aggressive clinical behavior. One of the most striking observations in our study is the inverse correlation between BIRC7 expression and DNA methylation at its promoter region. While global DNA hypomethylation has long been recognized in cancer biology, leading to chromosomal instability and oncogene activation, gene‐specific promoter hypomethylation remains relatively underexplored compared to promoter hypermethylation of tumor suppressors [28, 29]. Our findings add to the growing body of evidence that hypomethylation of oncogene promoters is a critical, yet underappreciated, event in thyroid tumorigenesis. Given that most studies have focused on silenced tumor suppressors in PTC (e.g., RASSF1A, SLC5A8), the identification of BIRC7 as a hypomethylated oncogenic target represents a significant paradigm shift. At the mechanistic level, we show that DNMT3a directly binds to the BIRC7 promoter and is essential for maintaining its methylation and transcriptional silencing. This aligns with prior studies that implicate DNMT3a in de novo methylation of developmental and cancer‐associated gene loci [30, 31]. However, unlike canonical models where DNMT expression is frequently downregulated in tumors, we found no significant change in DNMT3a mRNA levels between PTC and normal tissues. This prompted us to explore post‐translational regulation, leading to the identification of SUMOylation as a critical modulator of DNMT3a function.

SUMOylation has emerged as a key regulatory mechanism in chromatin biology, affecting transcription factor activity, chromatin remodeling complexes, and epigenetic enzymes [32]. Our demonstration that SUMO‐modified DNMT3a exhibits reduced chromatin binding at the BIRC7 promoter expands the functional repertoire of this modification beyond protein stabilization and nuclear localization. It suggests that SUMOylation serves as a negative regulatory mechanism that prevents DNMT3a to execute its repressive functions at specific genomic loci. These findings resonate with previous reports in hematologic malignancies where SUMOylation of DNMT3a affects target specificity and gene repression. In the context of solid tumors such as PTC, our work is among the first to highlight SUMOylation‐mediated epigenetic control as a determinant of oncogene regulation. The ability of SUMOylation inhibitors to enhance DNMT3a–BIRC7 interactions and derepress BIRC7 suggests that pharmacological targeting of the SUMO–DNMT3a–BIRC7 axis may have dual and possibly paradoxical outcomes. Therefore, systematically evaluating global SUMO levels across diverse thyroid tumor cohorts and correlating them with clinical staging represents a valuable avenue for future research.

Moreover, our findings raise important questions regarding epigenetic cooperativity in PTC. It remains to be determined whether other oncogenic loci are similarly regulated by SUMOylated DNMT3a, or whether BIRC7 represents a unique case of promoter‐specific control. Genome‐wide ChIP‐seq and methylome analyzes following SUMOylation inhibition would be valuable next steps to map the broader landscape of DNMT3a‐SUMO interactions. Additionally, it is plausible that other chromatin modifiers or transcriptional repressors interact with SUMOylated DNMT3a to orchestrate localized epigenetic silencing‐a hypothesis that warrants further investigation. Finally, the translational implications of our work are notable. Given the availability of clinically applicable SUMOylation inhibitors and DNMT‐targeting agents, BIRC7 expression and promoter methylation status may serve as predictive biomarkers for treatment responsiveness.

Lastly, several limitations of the current study should be acknowledged. While our findings convincingly demonstrate that the SUMOylation of DNMT3a is required to repress BIRC7 expression, PTC progression is an inherently complex processes that undoubtedly involves a multi‐gene and multi‐regulatory network. On one hand, besides SUMOylation, other potential post‐translational modifications (PTMs) of DNMT3a—such as phosphorylation, acetylation, or ubiquitination—might also be deficient or altered in carcinoma cells, potentially modulating its methyltransferase activity either synergistically or independently. On the other hand, SUMOylated DNMT3a likely coregulates a broader spectrum of target genes beyond BIRC7. Although conducting a global multi‐PTM mass spectrometry analysis or a genome‐wide ChIP‐seq screening was beyond the functional scope of this study, utilizing these high‐throughput technologies to comprehensively profile the modification landscape of DNMT3a and its genome‐wide targets will represent a critical direction for our future research.

## 5 Conclusion

In conclusion, this study elucidates a previously unrecognized SUMO‐DNMT3a‐BIRC7 regulatory circuit that integrates post‐translational and epigenetic mechanisms to control oncogene expression in thyroid cancer. By uncovering the functional significance of BIRC7 promoter methylation and its dependence on SUMOylated DNMT3a, we reveal new dimensions of epigenetic control in PTC and lay the groundwork for novel therapeutic interventions targeting epigenetic plasticity and apoptotic resistance.

## Author Contributions

Conceptualization and investigation: Kunpeng Liu and Hongguang Li. Methodology and resources: Zijie Su. Writing and original draft preparation: Kunpeng Liu and Hongguang Li. Critical review and editing: Kunpeng Liu and Hongguang Li. All authors contributed to editorial changes in the article. All authors have read and approved the final article. All authors have participated sufficiently in the work and agreed to be accountable for all aspects of the work.

## Ethics Statement

Informed consent was obtained from all participants included in the study. The study was approved by the Ethics Committee of the Henan Provincial People's Hospital, Zhengzhou University. All procedures related to the care and sacrifice of animals were reviewed and approved by the Ethics Committee of the Henan Provincial People's Hospital, Zhengzhou University and performed in accordance with international regulations (2022‐500‐177).

## Conflicts of Interest

The authors declare no conflicts of interest.

## Supporting information


**Figure S1: High‐resolution CpG‐level methylation analysis of the** ***BIRC7*** **promoter in the TCGA‐THCA cohort. A,** Schematic representation of the *BIRC7* locus on chromosome 20 (hg19), detailing the precise genomic coordinates of 13 CpG probes from the Illumina HumanMethylation450 (HM450K) array. Probes are categorized into a promoter cluster (8 CpGs) and a gene‐body cluster (5 CpGs). Node colors indicate differential methylation status between papillary thyroid carcinoma (PTC) and normal tissues (Blue: significantly hypomethylated in tumors, FDR < 0.05; Red: significantly hypermethylated in tumors, FDR < 0.05; Grey: not significant). **B,** Boxplots comparing the methylation levels (β‐values) of the 13 individual CpG probes between adjacent normal tissues (*n* = 56, blue) and PTC tumor tissues (*n* = 507, red). Statistical significance was evaluated using the two‐sided Mann‐Whitney U test with Benjamini‐Hochberg FDR correction (*FDR < 0.05, FDR < 0.01, ***FDR < 0.001, ****FDR < 0.0001, ns: not significant). **C,** Spearman correlation analysis between the methylation levels (β‐values) of the significantly hypomethylated promoter CpGs and *BIRC7* mRNA expression (log_2_ normalized counts) in the TCGA‐THCA cohort. Red lines represent the linear regression fit, with the shaded areas indicating the 95% confidence intervals. Strong negative correlations indicate that promoter hypomethylation drives *BIRC7* upregulation. **Figure S2: Genetic perturbation of the SUMO pathway via UBC9 knockdown reduces DNMT3a SUMOylation and BIRC7 expression. A,** Co‐immunoprecipitation (Co‐IP) assays evaluating the SUMOylation status of DNMT3a in BCPAP and K1 cells transfected with either negative control siRNA (siNC) or UBC9‐targeting siRNA (siUBC9). Cell lysates were immunoprecipitated with an anti‐SUMO1 antibody (or IgG negative control) and immunoblotted (IB) with an anti‐DNMT3a antibody to detect SUMOylated DNMT3a. The input panels show total DNMT3a and GAPDH levels. Representative images of three independent experiments are shown. **B,** Western blot analysis of BIRC7 protein expression in BCPAP and K1 cells following transient transfection with siNC or siUBC9. GAPDH was used as the loading control. Representative images of three independent experiments are shown. **C,** Quantitative real‐time PCR (qRT‐PCR) analysis of *BIRC7* mRNA levels in BCPAP and K1 cells treated with siNC or siUBC9. Knockdown of UBC9 significantly decreased *BIRC7* transcription compared to the siNC group in BCPAP (*p* = 0.0077) and K1 (*p* = 0.0012) cells. Data are presented as mean ± SD of *n* = 3 independent experiments. **Figure S3: Loss‐of‐function and epistasis analysis of the DNMT3a‐BIRC7 axis in PTC cells. A,** Cell viability was measured by CCK‐8 assays in K1 (top) and BCPAP (bottom) cells co‐transfected with BIRC7 shRNA (shBIRC7) and/or DNMT3a siRNA (siDNMT3a) over a 96‐hour period. Knockdown of BIRC7 significantly reduced cell viability compared to the control group (*p* = 0.0135 for K1; *p* = 0.0019 for BCPAP), whereas simultaneous knockdown of DNMT3a significantly rescued and reversed the BIRC7‐knockdown‐mediated suppression of cell growth (*p* < 0.001 for both K1 and BCPAP cells). Data are presented as mean ± SD of *n* = 3 independent experiments. **B,** Transwell invasion assays of BCPAP and K1 cells following single or double knockdown of BIRC7 and DNMT3a. Representative images of invaded cells are shown (top). Quantitative analysis (bottom) of invading cells per field demonstrates that BIRC7 depletion significantly inhibits cell invasion under control conditions (*p* = 0.0381 for BCPAP; *p* = 0.0186 for K1), which is prominently restored by concurrent knockdown of DNMT3a (*p* < 0.001 for both cell lines). Data are presented as mean ± SD of *n* = 3 independent biological replicates.

## Data Availability

The datasets generated during and/or analyzed during the current study are available from the corresponding author upon reasonable request.
